# Soft Self-Healing Fluidic Tactile Sensors with Damage Detection and Localization Abilities

**DOI:** 10.3390/s21248284

**Published:** 2021-12-11

**Authors:** Thomas George Thuruthel, Anton W. Bosman, Josie Hughes, Fumiya Iida

**Affiliations:** 1 Bio-Inspired Robotics Laboratory, Department of Engineering, University of Cambridge, Cambridge CB2 1PZ, UK; jaeh2@cam.ac.uk (J.H.); fi224@cam.ac.uk (F.I.); 2 SupraPolix BV, Horsten 1, 5612 AX Eindhoven, The Netherlands; bosman@suprapolix.com

**Keywords:** soft robotic sensors, self-healing sensors, fluidic sensing, damage detection

## Abstract

Self-healing sensors have the potential to increase the lifespan of existing sensing technologies, especially in soft robotic and wearable applications. Furthermore, they could bestow additional functionality to the sensing system because of their self-healing ability. This paper presents the design for a self-healing sensor that can be used for damage detection and localization in a continuous manner. The soft sensor can recover full functionality almost instantaneously at room temperature, making the healing process fully autonomous. The working principle of the sensor is based on the measurement of air pressure inside enclosed chambers, making the fabrication and the modeling of the sensors easy. We characterize the force sensing abilities of the proposed sensor and perform damage detection and localization over a one-dimensional and two-dimensional surface using multilateration techniques. The proposed solution is highly scalable, easy-to-build, cheap and even applicable for multi-damage detection.

## 1. Introduction

Self-healing elastomeric polymers can provide improved performances and novel functionalities to existing soft robotic and wearable systems [1,2,3]. They are primarily used for functional recovery of physical [4,5,6] and electrical properties [7,8,9]. Among them, self-healing soft sensory systems are of particular interest due to their potential applications [10]. However, there are numerous challenges in developing smart self-healing materials with the desired sensing properties while being able to repair-and-recover their functionality after a damage cycle in a fast and autonomous manner. This work presents a novel self-healing elastomer with rapid and autonomous self-healing capabilities combined with an intelligent fluidic sensing architecture. Our methodology allows us to develop fast-recovering self-healing soft sensors with tunable sensing properties and the ability to detect and localize damage in a continuous manner.

**Figure 1:**
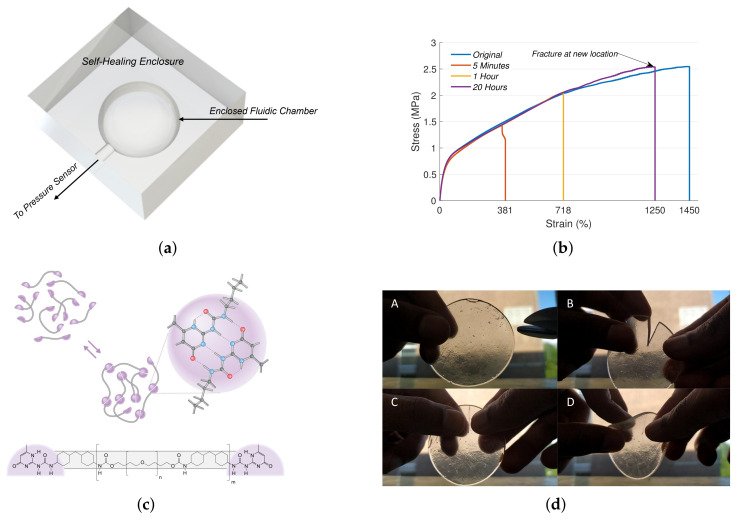
Working principle of the self-healing fluidic soft sensor. (**a**) Design of the fluidic sensor. The curvature of the chamber and the elasticity of the SH material ensures that the structural integrity of the fluidic chamber is maintained. (**b**) Healing characteristics of the SH elastomeric polymer at ambient conditions. Note that complete healing is not required for full functional recovery of the soft sensor. (**c**) Reversible hydrogen-bonding arrays responsible for the self-healing of the supramolecular polymer together with its molecular structure. (**d**) Instantaneous healing of the SH polymer under ambient conditions.

Soft robotic sensors are vital for obtaining information about internal physical states in a non-intrusive manner [11,12]. Typically, they are designed with materials that change their electrical properties in response to strain, stress, pressure, temperature, etc. [13]. Currently, self-healing soft sensors are made with composites of self-healing polymers and the addition of conductive metallic [14] or carbon particles [15,16,17]. There have been impressive demonstrations where 90% functional recovery was obtained after 15 s of healing at ambient temperatures [14]. Recent works have looked into improved sensing/healing properties [18,19,20], physical/optical properties [21], 3D printability [7] and bio-compatibility [22,23]. Nonetheless, the addition of conductive particles to the SH polymer leads to suboptimal mechanical and healing performances. Moreover, certain manual effort is required to bring damaged components together and align them. Here, we present a soft sensor design based on encapsulated fluid bodies that allows us to develop self-healing sensors with only the non-conductive SH polymers (see Figure 1a). This allows us to maintain the physical properties of the base SH polymer. Additionally, with the help of a supramolecular self-healing material, we can obtain almost instantaneous self-healing even at room temperature. This autonomous self-healing material consists of supramolecular polymers that comprise specific hydrogen-bonding arrays based on ureidopyrimidones [24], which combine high strength with a highly dynamic nature and therefore give self-healing properties to the materials [25,26]. Their ease of synthesis, processing and biocompatible nature make these supramolecular self-healing materials eminently suitable for their application in soft robotics [27]. With the appropriate design of the fluidic volume (highly concave internal surfaces), we can ensure that internal stresses created after damage will autonomously align and heal the soft sensor. As the sensors are made with only one material, fabrication also becomes easier. Similar design concepts have been used to develop soft sensors, albeit without the self-healing capabilities [28,29,30]. Due to the unique spectral characteristics of pressure signals in enclosed volumes that undergo damage and the relatively slow speed of pressure signals (speed of sound in the medium), our SH sensor design can be adapted to detect and localize damage in a continuous manner.

Damage detection abilities are vital for monitoring the integrity of the surrounding system and for safer interactions. Conventional methods have used specialized tools for damage detection and localization. They include techniques, such as visual inspection, X-ray radiography, ultrasonics, etc. [31,32,33,34]. However, these devices are specially designed for damage detection without additional sensing capabilities. Additionally, these methods are difficult to be transferred to soft-bodied systems in a cheap and practical way. Markvicka et al. have recently demonstrated a soft sensory skin that can detect and localize damage based on soft composite material with liquid metal droplets [35]. They demonstrate how such capabilities can facilitate an intelligent response to mechanical damages. However, their sensory skin did not have self-healing capabilities and requires a 2D array of parallel sensory fibers for damage detection and localization, which restricts their applicability and scalability. More recent works have looked into incorporating self-healing with damage detection and localization [36]; however, they still have all the disadvantages of composites of SH polymers and conductive particles. Moreover, the damage localization is discrete, requiring one sensor for detection of damage for each pre-defined location.

Continuous localization capabilities can be obtained by extending the proposed system by measuring the pressure signals inside the enclosed chamber through different pathways. Since pressure signals travel at the speed of sound in the medium, we can employ multilateration techniques for localizing the source of the pressure signal based on the time-of-flight of the pressure signal. As the pressure signals are generated by the contact or damage event itself, an additional energy source is not required for localization, unlike typical multilateration systems. Due to the impedance mismatch between the fluidic chamber and the surrounding self-healing material, the localization sensor can be scaled to any complex geometry.

This work presents a soft sensor design based on encapsulated fluid bodies that allows us to develop self-healing sensors with only the non-conductive supramolecular SH polymers. This allows us to obtain almost instantaneous self-healing even at room temperature. Due to the compliance of the material and its biocompatible nature, these sensors are well suited for soft robotic applications. We investigate the self-healing characteristics of the material experimentally and the sensing properties using a finite element model. Finally, we demonstrate the applicability of the sensor for damage detection and localization in a one-dimensional and two-dimensional sensing surface. This work is the first to demonstrate a soft self-healing sensor that can detect and localize damage on a continuous surface.

## 2. Materials and Methods

### 2.1. SH-Material and Fabrication

The self-healing supramolecular elastomer was obtained in a process described previously from 2 to amino-4-hydroxy-6-methyl-pyrimidine, 4,4,-methylenebis(cyclohexyl isocyanate) and poly(tetramethylene oxide) (Mn = 1000) [37], resulting in a telechelic polyurethane with ureidopyrimidone end groups and having a number average molar mass (Mn) of 20 kDa and a mass average molar mass (Mw) of 40 kDa (SEC in THF against PSt standards). The isolated polymer was subsequently processed into a clear film with thicknesses in the range of 0.5–1.0 mm by using a hydraulic laboratory press from Fontijne Press (Delft, the Netherlands) at 120 °C and 150 N.

Once the SH material is formed into films, the sensors can be developed using compression molding techniques (Figure 1a). The inverse mold of the sensor is 3D printed using heat resistance ABS. The SH films are placed on the mold, locally heated using a heat gun set at 100 degree Celsius and molded to the desired shape by applying pressure. Once the material is cooled down, the parts are removed from the mold. The connecting tube is added at this stage, and complementary parts can be attached by heating the open surfaces for a short duration and bring the surfaces together. This creates a leak-proof binding. The interface between the SH material and the non-SH connecting tube can have leaks due to impedance mismatch, in which case, the leaks are plugged using silicone glue. For both the damage detecting sensors, the non-SH connecting tube passes through the entire chamber for ease of fabrication and structural stability of the inner chamber. This also prevents the SH material from adhering onto itself during fabrication. As the connecting tube is thin, it does not affect the performance of the sensor after each damage-and-heal cycle.

### 2.2. Working Principle

The working principle of the sensor is based on the transmission of external forces through pressure waves inside the enclosed fludic chamber (Figure 1a). These pressure signals can be measured away from the contact location using a commercial pressure sensor. The morphology of the chamber determines the sensitivity of the sensor to the external stimulation, which is investigated in Section 3.1. The self-healing of the material happens because of the reversible hydrogen-bonds present in the polymer. Damage detection in the fludic chamber is feasible because of its characteristic frequency response. As damage is associated with a sharp drop is pressure, this creates pressure waves with a particular high frequency component. This component is a function of the chamber morphology. With high enough sampling frequnecy this component can be easily detected. Damage localization is conducted with multi-lateration techniques. As pressure waves travel at the speed of sound inside the chamber, by looking at the time-of-flight differences at two different pressure sensors, the location of the pressure source can be localized. We assume that the pressure waves travel along the shortest path with the effects of reflections neglected.

### 2.3. Experimental Setup

For measuring the pressure inside the chamber, we use the MPXH6400A Absolute, Integrated Pressure Sensor. The analog pressure values from the sensor are read using a National Instruments (NI) USB 6212 data acquisition system. The analog signals are then sampled at 200 KHz with a 16-bit resolution. For measuring the applied forces, we use the ATI Nano43 6-axis force sensor. The analog signals from the force sensors are amplified using the NI FTIFPS1 amplifier and then read by the NI USB-6212 ADC. The data from the data acquisition system is read through the serial port and processed in MATLAB. The indentation probe is controlled using a UR5 robotic manipulator.

### 2.4. Data Processing

All the data processing of the pressure signals is conducted on MATLAB. For characterizing the sensor properties, the sensor signals are read through the serial port on-demand. For detecting and localizing damage, the sensor signals are read continuously at 200 KHz for a total duration of 10 s. The raw signals are then filtered using a bandpass filter (using MATLAB bandpass function) with a passband frequency range of 150–1000 Hz for both the sensor morphologies. The steepness of the filter was set at 0.8, and the stopband attenuation was set to 50 dB. The filtered signal is further smoothened using a simple thresholding method to remove low decibel noise. The MATLAB function *finddelay* is used for measuring the lag between the two pressure signals.

For obtaining the pathway of the 2D sensor morphology, we use some standard image processing tools. First, a picture of the sensor is taken, and the pathway is manually traced on picture. A binary image of this is obtained after converting the picture to an HSV colormap. The MATLAB functions *imregionalmin* and *bwskel* are then used to trace the thinnest connected pathway in the binary image. This pathway in the image space is then calibrated with respect to the real system by using the total length of the fluidic tube and its corresponding pixel length.

## 3. Results

We study the capabilities of our proposed sensing system using three experimental scenarios. In the first scenario, we characterize the performance of the single-output fluidic sensor and investigate its potential as a force sensor. In the second and third experimental scenarios, we investigate and study the performance of the multi-output fluidic sensor for damage detection and localization in a one-dimensional and two-dimensional surface, respectively.

### 3.1. Sensor Characterization

**Figure 2:**
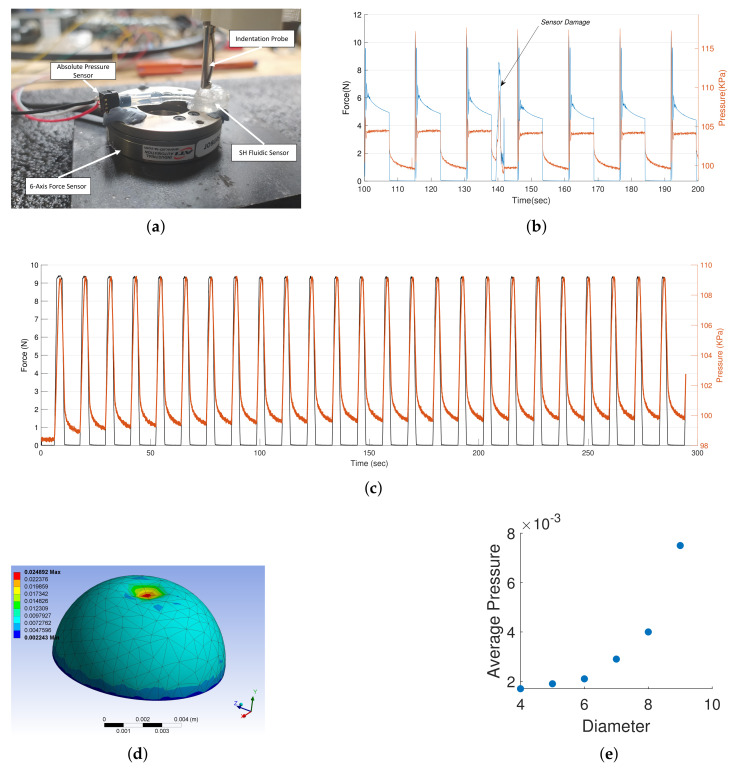
The single output hemisphere sensor characterization. (**a**) Experimental setup for characterizing the single output self-healing soft sensor. (**b**) Sensor response to damage during a periodic step indentation. (**c**) Sensor response to a periodic truncated sine signal. (**d**) Ansys model for investigating the relation between sensor geometry and force sensitivity. (**e**) Exponential relation between the diameter of the hemisphere and the internal pressure for a constant force input.

The single-output hemispherical sensor schematically represented in Figure 1a can be used as a simple soft force sensor. It must, however, be pointed out that being a soft sensor, the fluidic sensor will respond to multiple physical cues. Hence, discerning the applied force in one direction using a single sensor is not possible without simplifying assumptions [12,38]. For practical applications, a large array of these sensors are required to decouple and estimate applied forces without constraints. The setup used for measuring the response of the soft sensor is shown in Figure 2a. The fluidic chamber is a hemisphere of 6 mm diameter, and the enclosing surface is a cuboid of dimension 10 mm × 10 mm × 5 mm. The pressure response of the sensor to a periodic indentation to constant height is shown in Figure 2c along with the measured vertical forces. We can see that the sensor response to the applied force is highly repeatable and the pressure response is in sync with applied force, at least during the application of force. Upon removal of the load, there appears to be a slow return to the baseline pressure due to the viscous effects of the material, which delays the return of the sensor to its original geometry. There is also a drift in the baseline pressure possibly due to strain relaxation in the material. These temporal nonlinearites can however be compensated by recent advancements in learning-based techniques [38,39]. By tuning the geometric parameters of the sensor, the response of the sensor to the applied force can be tuned. The relation between applied force to the measure pressure is analyzed using an Ansys model (Figure 2d), and the results are shown in Figure 2e. As the diameter of the hemisphere increases with respect to the enclosing surface, we can obtain higher sensitivity to applied vertical forces. This sensitivity is independent of the material stiffness, viscosity and Poisson’s ratio as verified through the simulation.

Finally, the resilience of the sensor to damage is studied. For this, we apply constant and impulsive indentation to the sensor. Impulsive forces are used to ensure that the enclosure is tightly sealed after damage. The results from this test are shown in Figure 2b. Upon first contact, we can see a sudden spike in the pressure and force value due to the impulse forces. Small oscillations can also be observed due to the oscillations of the robotic arm to which the indenter is attached to. For this step signal, we can see that the internal pressure quickly settles to a constant, while the applied force on the force sensor slowly settles to a constant. This indicates that the stress relaxation occurring in the material while the force is applied does not change the geometry of the deformed chamber; hence, the pressure remains constant. The sensor is damaged using a surgical knife around the 140 s mark. We can observe that on the next indentation, the pressure is still maintained at a constant value even though the material had only few seconds to heal. No indication of a leak is observed here. There is however a small shift in the peak pressure, indicating that an additional calibration process might be required after damage.

### 3.2. One-Dimensional Damage Detection and Localization

**Figure 3:**
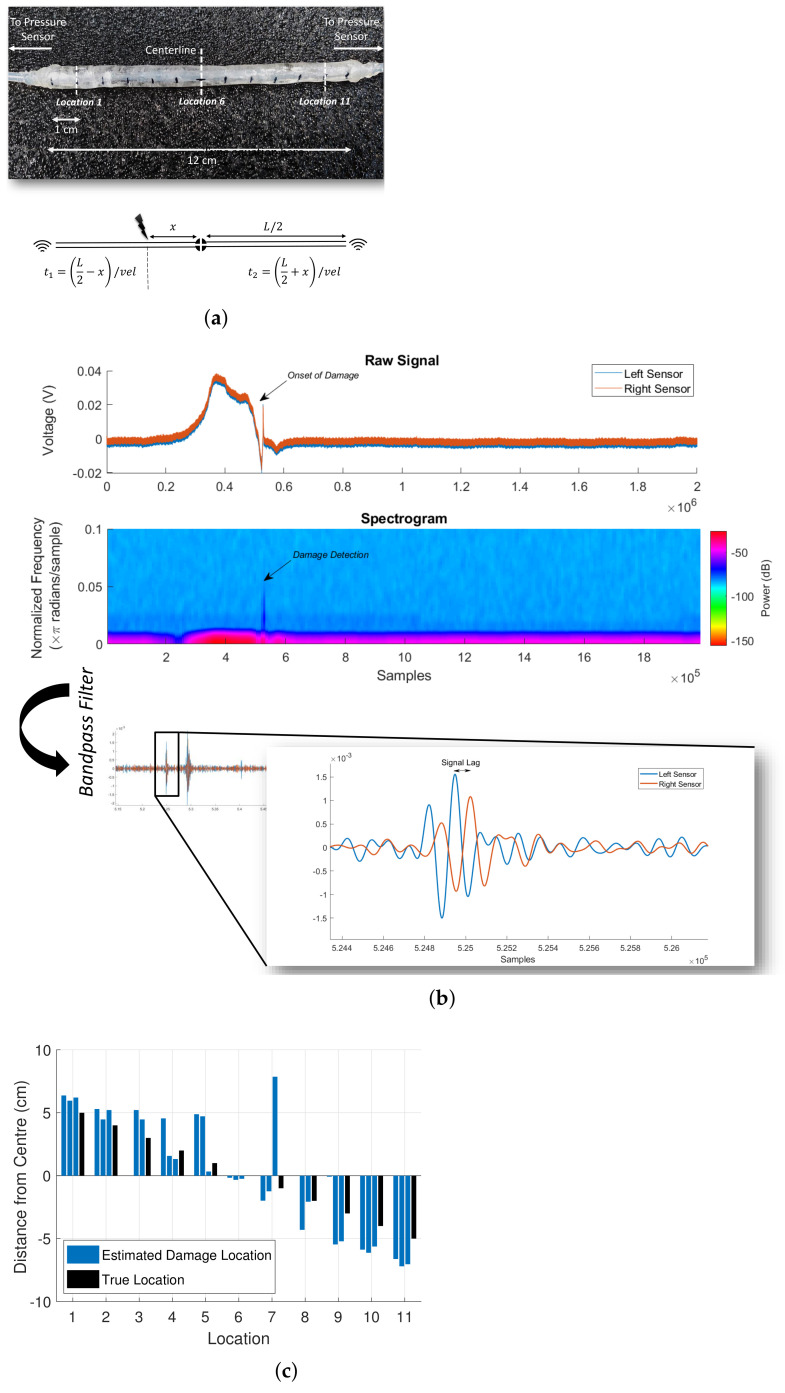
One-dimensional damage detection and localization using the multi-output fluidic sensor. (**a**) One-dimensional damage detection and localization sensor. The time-of-flight model for the 1D model is shown below. (**b**) Processing of the raw pressure signals for damage detection and localization. (**c**) Damage localization accuracy of the one-dimensional damage sensor.

The damage detection and localization capabilities of the multi-output self-healing sensor is investigated along one dimension first. The sensory system and its schematic is shown in Figure 3a. Any external contact on the sensor creates pressure waves that travel at the speed of sound, originating from the point of contact. The pressure waves will travel along the path of the least resistance. The signals are severely attenuated when it travels from one medium to the other. Hence, by measuring the time difference between the arrival of the pressure signal at the two pressure sensors and knowing the path of the pressure signal, we can triangulate the location of contact. The time-of-arrival difference ($t_{2}-t_{1}$) can be obtained as:

$$t_{2}-t_{1}=\frac{L/2+x}{vel}-\frac{L/2-x}{vel}$$

$$t_{2}-t_{1}=\frac{2x}{vel}$$

where *L* is the total length of the air cavity between the ends of the two pressure sensors, *x* is the distance of contact from the centre of the air cavity, and vel is the speed of sound in the medium. For all our experiments, we use the speed of sound as 33,100 cm/s, which is the speed of sound at zero degrees Celsius. For the resolution of our setup (0.165 cm), a 100% error in the estimate of the sound speed would only lead to an error bias of 0.165 cm.

**Figure 4:**
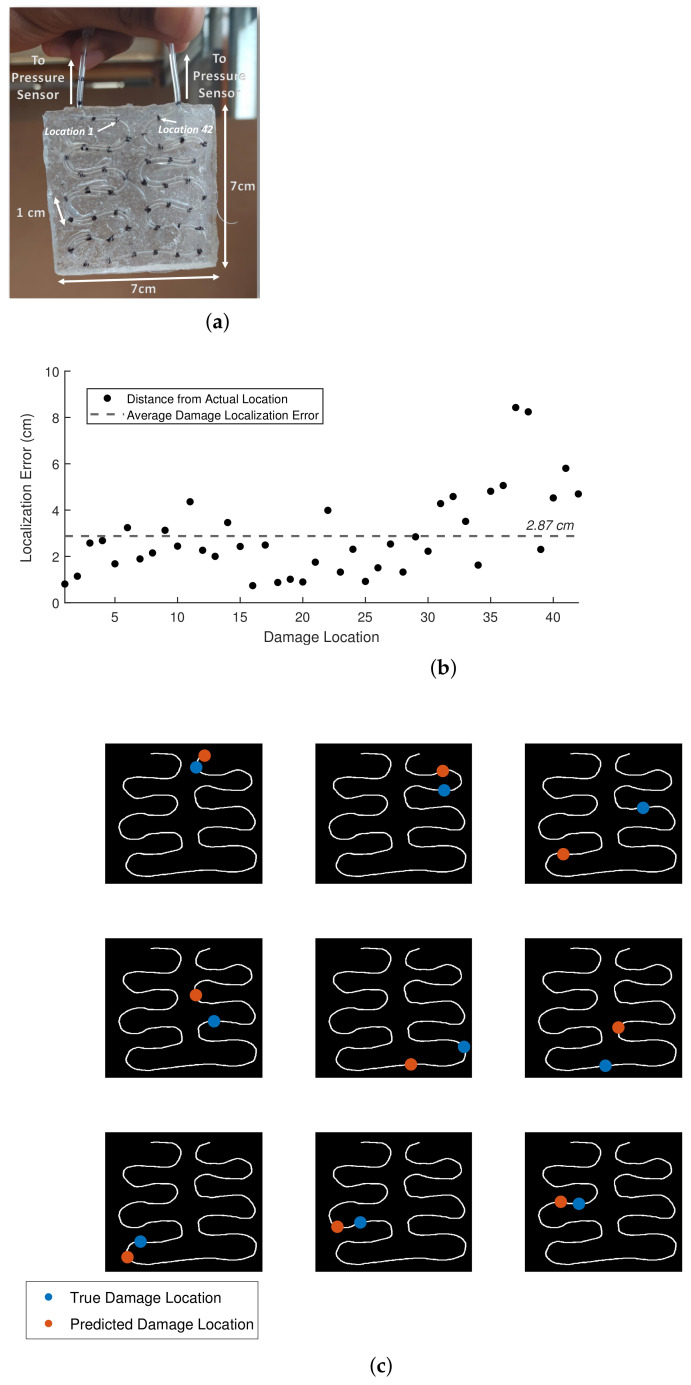
Two-dimensional damage detection and localization using the multi-output fluidic sensor. (**a**) The 2D sensory structure for continuous damage detection and localization along a surface area. (**b**) Localization error at each location of damage. (**c**) Examples of damage detection and location for the 2D sensor morphology.

Measuring the time-of-arrival difference requires the identification of discernible features in the signal for the particular sampling frequency. As the sampling frequency decreases, the features must lie at a higher frequency spectrum for accuracy. Hence, normal contacts are difficult to be localized. However, damage events have high-frequency components that are characteristic of their internal geometry. This makes the detection and localization easier even at a relatively low sampling frequency of 200 KHz. For our sampling rate, the highest localization resolution we can obtain is 0.165 cm. The raw pressure signals and its spectrogram are shown in Figure 3b. When the sensor is damaged, a high-frequency component can be observed. Due to noise in the raw pressure signal, detecting features for the time-of-arrival difference measurement is not possible. Therefore, the raw signal is filtered with a bandpass filter whose range is manually estimated for each sensor geometry. Once the filter parameters are fixed, damage can be reliably detected, irrespective of the damage location. Detecting features in the filtered signal is now easier (see Figure 3b). To test the performance of our damage detection and localization setup, we perform an experiment where the sensors are damaged in known locations along the sensor length. There were a total of 11 locations, and each location was damaged thrice. The results are shown in Figure 4b. Of the 33 damages, 31 of the damages were detected, amounting to a detection accuracy of 93%. The localization accuracy was 1.13 ± 1.41 cm. Non-detection of damage happens when the filtered signals are too weak. Using more sensitive pressure sensors with a low signal-to-noise ratio will greatly improve the damage sensing abilities and also allow normal contact localization abilities. Alternatively, having a thicker SH matrix will improve the damage detection ability, as higher power is required to induce damage in that case.

### 3.3. Two-Dimensional Damage Detection and Localization

As mentioned before, due to the high impedance among different mediums, the path taken by the pressure signal is almost always along the fluidic chamber, even if the chamber is not straight. This allows us to easily scale the proposed system to arbitrarily complex surfaces with the same number of output pressure sensors. Figure 4a shows the design of a SH sensory structure for 2D damage detection and localization. The total length of the fluidic chamber is around 42 cm. Similar to the previous subsection, the sensory skin is manually damaged at marked locations, and the damage localization error is measured. Due to the complex morphology of the fluidic path, the 2D path of the chamber is estimated using computer vision techniques. Each marked location was damaged twice. Due to the higher thickness of the current system, all the damage instances were detected. The localization error was slightly higher at 2.87 ± 2.26 cm. This amounts to a localization error within 15% of the length of the sensory pathway. The absolute error at each location is shown in Figure 4b. Examples of damage localization on the 2D morphology for nine random points are shown in Figure 4c. Although we assume that the pressure waves travel along the fluidic chamber due to the curvature of the shape, there are possible reflections that might affect the time-of-arrival difference estimation.

## 4. Conclusions

This article presents the design and fabrication of a self-healing soft fluidic sensor. The sensor works on the principle of information transfer from the physical stimuli via pressure waves traveling in an enclosed fluidic chamber. This allows us to obtain tactile information remotely and unobtrusively. More importantly, this enables us to fabricate these soft sensors without the addition of any functional materials. By using self-healing supramolecular polymers as the enclosing matrix, we obtain exemplary self-healing properties characterized by instantaneous functional recovery at ambient conditions without external inputs. We perform extensive characterization of the soft sensor and its application as a force sensor. Furthermore, using finite element analysis, we demonstrate how the chamber morphology can be designed to obtain higher sensitivity to applied forces. By leveraging acoustic characteristics of damage and the relatively slow speed of pressure waves, we finally show how our sensing principle can be used to even detect and localize damage in a continuous manner indefinitely. To the best of our knowledge, this is the first demonstration of such a use of a soft sensor. We demonstrate highly reliable damage detection and accurate localization abilities on our modified multi-output sensor morphologies using commonly available hardware. The detection and localization performance can be improved and conducted in real-time by executing all the computation using electronic components. This would also facilitate highly scalable contact localization abilities using just two output pressure sensors. The ability to detect and localize damage repeatedly could be an important tool for soft robots and soft wearable devices for monitoring structural integrity and as a feedback mechanism to adapt and react to damage-causing actions. These systems can also be devised for structural health monitoring as a replacement to conventional Non-Destructive Testing techniques due to low compliance, ease of manufacturing and deployment. The self-healing capabilities also increase the life-span of the sensors.

## Data Availability

Supporting Information will be available upon request.
